# Mitophagy suppression via lncRNA H19 silencing: a novel strategy to overcome cisplatin resistance in lung adenocarcinoma

**DOI:** 10.1080/15384101.2025.2581634

**Published:** 2025-11-05

**Authors:** Meng-Zhen Liu, Xiao-Yan Shao, Si-Han Wu, Qi-Qi Ning, Can Zhang, Wei-Wei Du, Rong-Rong Sun, San-Yuan Sun, You-Wei Zhang

**Affiliations:** a Department of Medical Oncology, Southeast University Affiliated Xuzhou Central Hospital, Xuzhou, China; b Department of Medical Oncology, Xuzhou Central Hospital, Clinical School of Xuzhou Medical University, Xuzhou, China; c Department of Internal Medicine, Zhumadian Central Hospital, Zhumadian, China; d Department of Molecular Microbiology and Immunology, University of Southern California Keck School of Medicine, Los Angeles, CA, USA

**Keywords:** lncRNA H19, LUAD, cisplatin resistance, mitophagy

## Abstract

Cisplatin (DDP) resistance substantially compromises treatment efficacy in lung adenocarcinoma (LUAD). This study investigates the role of mitochondrial long non-coding RNA (lncRNA) H19 in mediating DDP resistance. High-throughput sequencing and RT-qPCR analyses revealed pronounced H19 upregulation in DDP-resistant A549 (A549/DDP) cells relative to parental A549 cells. Subcellular localization studies indicated that H19 is primarily nuclear in A549 cells but translocates to mitochondria in A549/DDP cells. Functional assays demonstrated that H19 silencing in resistant cells attenuated chemoresistance, suppressed proliferation, migration, invasion, and colony formation in vitro, and delayed tumor growth in vivo. H19 knockdown impaired mitophagy and promoted apoptosis, mirroring autophagy inhibition and restoring DDP sensitivity. In contrast, H19 overexpression in A549 cells did not significantly alter mitophagy or cellular behavior. Furthermore, H19 silencing induced its relocalization from mitochondria back to the nucleus in resistant cells, while overexpression did not affect its nuclear localization. These findings establish that H19 translocation to mitochondria promotes DDP resistance, and its downregulation reverses this process by inhibiting mitophagy and resensitizing cells to DDP. As a nucleus-encoded mitochondria-associated lncRNA (ntmtlncRNA), H19 mediates intercompartmental communication, highlighting its potential as a therapeutic target for overcoming DDP resistance in LUAD.

## Introduction

Lung cancer continues to pose a significant global health burden in 2024, with the United States expected to have the second highest incidence and the highest mortality rate for both sexes [1]. In contrast, data from 2022 indicate that China currently has the highest global burden, with approximately 1,060,600 new cases and 733,300 deaths [2]. Non-small cell lung cancer (NSCLC) constitutes approximately 85% of all lung cancer diagnoses, and lung adenocarcinoma (LUAD) is its most prevalent subtype [3]. Platinum-based chemotherapy, particularly cisplatin (DDP), remains a cornerstone of LUAD treatment [4–6]. Although immunotherapies – such as inhibitors targeting PD-1/PD-L1 and CTLA-4—have transformed cancer management, conventional chemotherapy continues to play an indispensable role in NSCLC treatment protocols [7–9]. Therefore, understanding the molecular basis of DDP resistance and developing strategies to enhance chemosensitivity are essential for improving clinical outcomes.

Mitochondria are centrally involved in mediating DDP resistance. The cytotoxic effects of DDP primarily result from DNA binding and the induction of apoptosis [10]. Tumor cells, however, can evade this cell death pathway through various mitochondrial mechanisms, including alterations in oxidative phosphorylation, enhanced antioxidant defense, modulation of oxidative stress, mitochondrial DNA mutations, shifts in membrane potential, and changes in mitochondrial dynamics [11–15]. As critical regulators of metabolism and apoptosis, mitochondria influence chemotherapeutic responses by controlling energy production, maintaining redox balance, regulating reactive oxygen species (ROS), and activating the mitochondrial permeability transition pore (mPTP) [16]. These mitochondrial processes are vital for tumor survival and chemoresistance, underscoring the importance of elucidating their regulatory mechanisms.

Mitochondria-associated long non-coding RNAs (lncRNAs) have recently emerged as key regulators of chemoresistance in cancer. For example, the lncRNA HOTAIR promotes chemoresistance by enhancing hexokinase 2 (HK2)-driven glycolysis and facilitating mPTP opening. Specifically, HOTAIR increases HK2 expression by sponging miR-125, thereby suppressing glioblastoma cell proliferation and promoting temozolomide-induced apoptosis [17]. Similarly, inhibition of lncRNA16 increases chemosensitivity in lung cancer through the HBB/NDUFAF5/ROS pathway [18]. The p53-responsive lncRNA neuroblastoma associated trans-cript 1 (NBAT1) also modulates drug sensitivity in aggressive neuroblastoma [19]. Previous studies have shown that H19 can repress the expression of pro-apoptotic genes, resulting in decreased levels of BAX and BAK proteins, which are required for cytochrome c (CytC) release and caspase activation [20,21]. Furthermore, H19 has been associated with the development of drug resistance in colorectal cancer, malignant melanoma, and LUAD [22–24]. Despite these advances, the roles of lncRNAs in mitochondrial-associated chemoresistance remain poorly understood, warranting further investigation.

In this study, we found that H19 was primarily localized in the nucleus of A549 cells, whereas in DDP-resistant cells, it was predominantly located in the mitochondria. As a nucleus-encoded mitochondria-associated lncRNA (ntmtlncRNA) [25], H19 dynamically shuttles between the nucleus and mitochondria, where it critically regulates mitophagy and promotes DDP resistance in LUAD cells. These findings offer new insights into the previously uncharacterized role of H19 in DDP resistance and suggest potential therapeutic strategies for improving chemotherapy efficacy in lung adenocarcinoma.

## Methods

### Cell culture and transfection

The A549 and A549/DDP cell lines were obtained from the Central Laboratory of Xuzhou Central Hospital. Lentiviral vectors were constructed by Shanghai Jikai Gene Medical Technology Co., Ltd. Both cell lines were maintained in high-glucose DMEM supplemented with 10% fetal bovine serum and incubated at 37°C with 5% CO₂. Cells in the logarithmic growth phase were harvested and resuspended at a density of 1.5 × 10^4^ cells/mL, then seeded into 96-well plates at 100 µL per well. Transfection was performed 24 hours after seeding. The experimental design included the following groups: an empty vector control (A549-NC and A549/DDP-NC), an experimental group (A549-OE-H19 and A549/DDP-sh-H19, where NC denotes negative control and sh-H19 indicates short hairpin RNA targeting H19), and a blank control group. Stable cell lines were established 48 hours post-transfection and used for subsequent experiments.

### Cell counting kit-8 (CCK-8) assay

Cells were harvested during the logarithmic growth phase, digested with trypsin, and centrifuged at 1000 rpm for 5 minutes at 4°C. The resulting pellet was resuspended to generate a single-cell suspension. Cells were then seeded into 96-well plates at a density of 5 × 10^3^ cells per well and incubated overnight at 37°C in a 5% CO₂ atmosphere. After confirming cell attachment the following day, the culture medium was replaced with fresh medium containing varying concentrations of DDP (0–80 μM). Each experimental condition included three replicate wells, and all assays were performed in three independent experiments. Following 48 hours of drug exposure, CCK-8 reagent (ServiceBio, G4103) was added to each well at a 1:10 dilution in culture medium, and the cells were incubated for an additional hour at 37°C. Absorbance was measured at 450 nm using a microplate reader. For proliferation assays, the same procedure was repeated with DDP-treated cells incubated for 0, 24, 48, and 72 hours before CCK-8 addition and absorbance measurement.

### Mitochondrial isolation, mitochondrial protein and rna extraction

Mitochondria were isolated using a Cellular Mitochondrial Separation Kit (Beyotime, NG228S). Following cell counting, cells were resuspended in Mitochondrial Separation Reagent (supplemented with PMSF when protein extraction was intended) and incubated on ice for 20 minutes. The cell suspension was subsequently homogenized with approximately 30 strokes using a glass homogenizer. Mitochondrial fractions were purified through density gradient centrifugation according to the manufacturer’s protocol. For mitochondrial protein extraction, the mitochondrial pellet was treated with 150 μl of lysis buffer containing 2 μl of PMSF, incubated on ice for 20 minutes with vortexing at 5-minute intervals, and then centrifuged at 12,000 × g for 5 minutes at 4°C. The resulting supernatant was collected for subsequent analysis. For mitochondrial RNA extraction, mitochondria were lysed using RNA-easy isolation reagent (Vazyme, R701). The lysate was combined with 400 μl of RNase-free water, incubated for 5 minutes, and centrifuged at 12,000 × g for 15 minutes. The aqueous phase was transferred to a new tube and mixed with an equal volume of isopropanol. After incubation at −20°C for 20 minutes and −80°C for 5 minutes, the sample was centrifuged again. The supernatant was discarded, and the RNA pellet was washed with 75% ethanol, followed by centrifugation at 8,000 × g for 5 minutes at 4°C. The pellet was air-dried and finally resuspended in 20 μl of RNase-free water, vortexed thoroughly, and stored at −80°C for future use.

### RT-qPCR and rna sequencing

The purity of isolated mitochondrial fractions was assessed through RT-qPCR and mitochondrial RNA sequencing. For RT-qPCR analysis, mitochondrial RNA was reverse transcribed into cDNA and amplified using SYBR Green qPCR Master Mix (ServiceBio, G3328) on a StepOnePlus™ Real-Time PCR System (Applied Biosystems). The relative expression levels of mitochondrial-encoded gene MT-ND5 and cytoplasm-localized GAPDH were measured to evaluate mitochondrial enrichment, with calculations performed using the 2^-ΔΔCT method. Several lncRNAs – including LINC00998, H19, MMP24-AS1, LINC01133, FLG-AS1, and LINC00707—were validated by this approach. Primer sequences for target genes and the mitochondrial reference gene MT-ND5 are provided in Table 1, while amplicon sizes, annealing temperatures, and GenBank Accession numbers are summarized in Table S1. Additionally, high-throughput mitochondrial RNA sequencing was conducted by Shanghai Inverse Ear Biotechnology Co., Ltd. (Shanghai, China) for comprehensive transcriptome profiling.

**Table 1.** Primers sequences for RT-qPCR.

Genes	Forward primer (5”−3”)	Reverse primer (5”−3”)
MTND5	ATGGCACATGCAGCGCAAGTAGGTC	GTTAGGAAAAGGGATACAGGAC
H19	CTCCCAGAACCCACAACATG	CAGTGGTTGAAAGTGCAGCATA
LINC00998	TGCCTGTTGTGGAAGCAGTAGAA	GCACAAGGCAGGCAAGACCA
MMP24-AS1	ACGAVGTGCGCTTCCTCAT	AGACAAGGCAGGCAAGAACCA
LINC01133	AACCTTTGCTCCAACTTTCTCCT	CTCTTTACCTCCTCCCAACCATT
FLG-AS1	TGTCCCTCACTGTCCCTGTCCT	GTCTCCCTCTGTGACTTCCCTCT
LINC00707	GACTTTACTGGCTTTCTTGCTCC	GACCTTAACCTTCCATCATCCCT

### Western blotting analysis

Cellular proteins were extracted using RIPA lysis buffer (Beyotime, P0013B), and protein concentrations were determined with a BCA assay kit (ServiceBio, G2026). Proteins were separated by 10% SDS-PAGE (Beyotime, P0012AC) with electrophoresis performed at 80 V for 30 minutes followed by 120 V for 60 minutes. PVDF membranes (Beyotime, P0965) were activated in methanol and assembled in a transfer cassette with sponge pads, filter paper, and the membrane. Protein transfer was conducted using a wet transfer system, with transfer duration optimized according to the molecular weight of each target protein. Following transfer, membranes were blocked for 1 hour at room temperature and subsequently incubated with primary antibodies overnight at 4°C. After three washes with TBST, membranes were incubated with corresponding secondary antibodies for 1.5 hours at room temperature, washed three additional times with TBST, and then developed using enhanced chemiluminescence reagent (ServiceBio, G2019). Signal detection was performed using a Bio-Rad imaging system. Primary antibodies against COXIV, GAPDH, caspase3, caspase9, Parkin, CytC, cleaved caspase3, cleaved caspase9, TOMM20, p62, LC3B, and LAMP2 were purchased from ServiceBio, while the PINK1 antibody was obtained from Abcam. Original western blot images for all experiments are provided in Figure S1 and correspond to the presented results.

### Fluorescence in situ hybridization (fish) assay

A fluorescence in situ hybridization (FISH) probe targeting H19 (sequence provided in Table S2) was synthesized by Wuhan Xavier Biotechnology Co. Cells were grown on coverslips in 24-well plates and fixed with 4% paraformaldehyde for 15 minutes at room temperature. Permeabilization was performed using 0.5% Triton X-100 at 4°C for 5 minutes. Following pre-hybridization in appropriate buffer at 37°C for 30 minutes, cells were hybridized with the probe overnight at 37°C in a dark humidified chamber. Post-hybridization, cells were washed with hybridization wash solution at 42°C in the dark, followed by a final PBS wash. Nuclei were counterstained with DAPI for 10 minutes and washed with PBS. Coverslips were mounted onto slides using fluorescence mounting medium, and fluorescence signals were visualized using a fluorescence microscope.

### Plate scratching experiment

A standardized wound healing assay was performed to assess cell migration capacity. Cells were seeded in six-well plates and cultured until they reached logarithmic growth phase and formed a confluent monolayer. Using a sterile pipette tip, a uniform linear scratch was created through the cell monolayer. The dislodged cells were gently removed by washing with PBS, and fresh serum-free medium was added to eliminate the influence of cell proliferation. The initial wound gap (0-hour time point) was immediately documented using microscope imaging. Following 24 hours of incubation under standard culture conditions, the same predefined fields of view were recaptured to evaluate cell migration into the wound area. The extent of wound closure was quantified by measuring the reduction in gap width using image analysis software.

### Plate cloning experiment

Cells were seeded in six-well plates at a density of 600 cells per well to assess clonogenic survival. After 24 hours of incubation, cells were treated with DDP for an additional 24 hours before the culture medium was replaced. The medium was subsequently refreshed every three days over a 15-day period to allow for colony development. Following the incubation period, cells were washed with PBS, fixed with 4% paraformaldehyde for 15 minutes, and stained with crystal violet solution for 10 minutes. After thorough washing to remove excess stain, the resulting colonies were documented by photographic imaging. Colonies consisting of more than 50 cells were counted to determine clonogenic capacity.

### Transwell experiment

For the migration assay, a cell suspension was prepared at a density of 1 × 10^6^ cells/ml, and 100 μl was added to the upper chamber of each transwell insert. The lower chamber was filled with 600 μl of complete medium containing the treatment compounds. After 24 hours of incubation, the inserts were carefully removed and rinsed twice with PBS. Cells were fixed with 4% formaldehyde for 15 minutes and stained with 0.1% crystal violet for 10 minutes. Non-migrated cells on the upper surface of the membrane were gently removed using a cotton swab. The inserts were air-dried and visualized under a microscope for imaging and quantification. For the invasion assay, transwell inserts were pre-coated with 100 μl of diluted Matrigel and incubated at 37°C for 3 hours to allow gel formation. Cells were pre-incubated in serum-free medium for 30 minutes before being seeded into the Matrigel-coated upper chambers. The subsequent steps, including incubation, fixation, staining, and cell removal, were performed identically to the migration assay protocol described above.

### Nude mouse experiment

Female BALB/c-nu nude mice (4–6 weeks old) were obtained from the Animal Center of Xuzhou Medical University and housed in a specific pathogen-free (SPF) environment. All experimental procedures were approved by the Institutional Animal Care and Use Committee of Xuzhou Medical University and conducted in accordance with ARRIVE guidelines. The mice were randomly divided into three groups (*n* = 6 per group) using a computer-generated randomization protocol. Tumor xenografts were established by subcutaneous inoculation of cells into the left forelimb of mice in the respective groups. After seven days, when palpable tumors had formed, mice received intraperitoneal injections of DDP (5 mg/kg) every three days for a total of three administrations. Tumor dimensions were measured every three days using digital calipers, and tumor volumes were calculated using the formula: V = (a × b^2^)/2, where “a” represents the longest diameter and “b” the perpendicular shorter diameter. Tumor growth curves were generated based on these measurements. Three weeks after initiation of treatment, all mice were euthanized according to established humane endpoints. Tumors were carefully excised, photographed, weighed, and processed for subsequent TUNEL staining to assess apoptotic activity.

### TUNEL and Immunofluorescence staining

Paraffin-embedded tumor sections were deparaffinized in xylene and rehydrated through a graded ethanol series. After washing with PBS, a hydrophobic barrier was traced around each tissue section using a liquid blocker pen. Sections were then treated with 100 μl of proteinase K (20 μg/ml) at 37°C for 15 minutes to expose DNA fragments. Following PBS rinses, 50 μl of TUNEL assay reaction mixture (ServiceBio, G1504) was applied to each section, which were subsequently incubated at 37°C for 60 minutes in a humidified dark chamber. Slides were coverslipped using fluorescence-compatible mounting medium and examined under a fluorescence microscope. Apoptotic cells were identified by positive green fluorescence staining, with appropriate positive and negative controls included in each assay batch.

### Mitochondrial membrane potential JC-1 assay

Cells in the logarithmic growth phase were harvested, and approximately 3 × 10^6^ cells were resuspended in 0.5 mL of culture medium. An equal volume of JC-1 staining working solution (ServiceBio, G1515) was added, and the mixture was gently inverted to ensure thorough staining. The cells were then incubated at 37°C for 20 minutes. Following incubation, the cells were centrifuged at 600 × g for 4 minutes at 4°C. The supernatant was carefully discarded, and the cell pellet was resuspended in 1 mL of JC-1 staining buffer (1×). Samples were transferred to black 96-well plates and analyzed using a fluorescence spectrophotometer.

### Statistical analysis

All statistical analyses and data visualization were conducted using GraphPad Prism version 8.0. Comparisons between two independent groups were performed using unpaired Student’s t-test for normally distributed data, while the Mann-Whitney U test was applied for non-normally distributed data. For comparisons among multiple groups, one-way ANOVA followed by Tukey’s post-hoc test was used for normally distributed data, with non-parametric alternatives employed when data violated normality assumptions. A p-value of less than 0.05 was considered statistically significant. All data presented are representative of three independent biological replicates, with values expressed as mean ± standard deviation unless otherwise specified.

## Results

### Differential expression of mitochondrial lncRnas in A549 and A549/DDP cells

**Figure 1.**
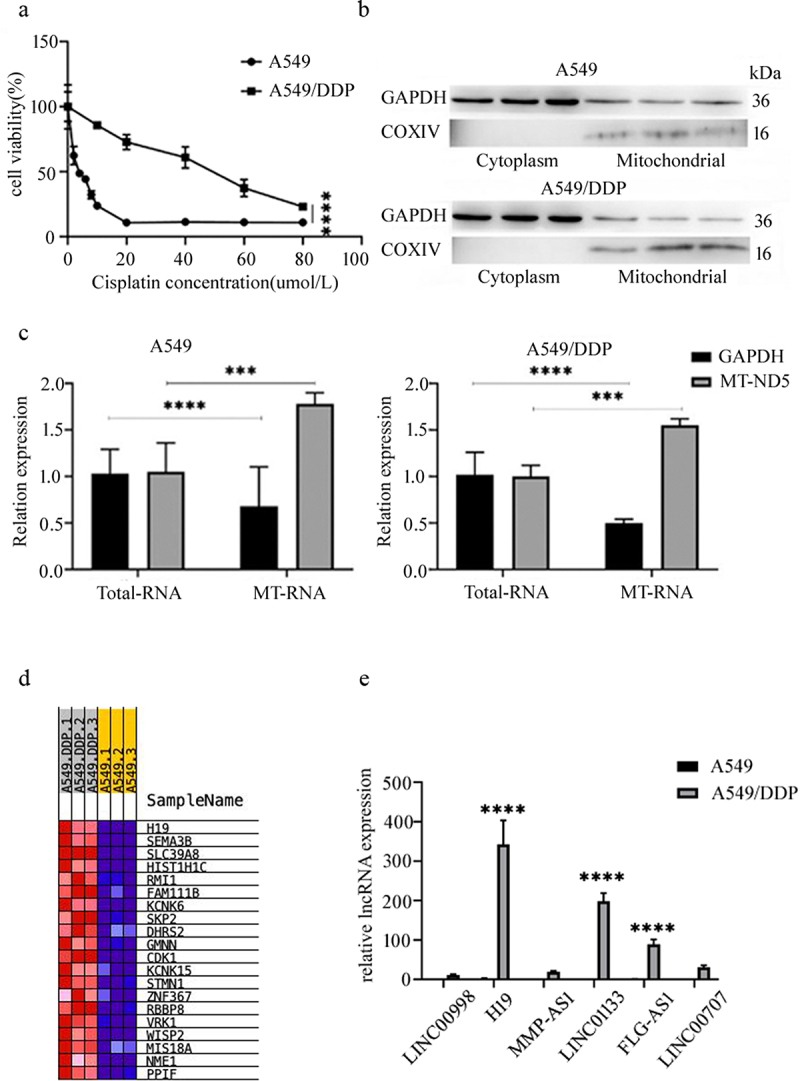
Differential expression of mitochondrial lncRnas in A549 and A549/DDP cells. (a) CCK-8 determination of resistance index of A549/DDP. Results represent three independent experiments and are expressed as sd ± mean (bars). Differences in means between the two samples were analysed by the the nonparametric rank sum test. (b) Validation of mitochondrial mass of A549 and A549/DDP cells. Results represent three independent experiments. (c) RT-qPCR detection of mitochondrial rna purity in A549 and A549/DDP cells. Results represent three independent experiments and are expressed as SD ± mean (bars). Differences in means between the two samples were analysed by the one-way anova. (d) Heat map of H19 by high-throughput sequencing, red represents high expression of genes in mitochondria, while blue represents low expression. (e) Real-time quantitative pcr to verify the expression of relevant lncRnas in cellular mitochondria.Three replicate samples were set up for each set of experiments and all experiments were repeated three times independently. Results represent three independent experiments and are expressed as SD ± mean (bars). Differences in means between the two samples were analysed by the t-test. ***p < 0.001; ****p < 0.0001.

The CCK-8 assay demonstrated that cell viability decreased with increasing DDP concentrations in both A549 and A549/DDP cell lines; however, A549/DDP cells maintained significantly higher viability, confirming their resistant phenotype (Figure 1(a)). Mitochondrial fractions were isolated and their purity was validated by western blot analysis using COX IV as a mitochondrial marker and GAPDH as a cytoplasmic marker (Figure 1(b)). The integrity of mitochondrial RNA was verified by comparing the relative expression levels of the cytoplasmic gene GAPDH and the mitochondrial-encoded gene MT-ND5 in total versus mitochondrial RNA fractions using RT-qPCR (Figure 1(c)). High-throughput mitochondrial RNA sequencing revealed the ten most abundantly expressed lncRNAs, among which H19 showed markedly different expression patterns between mitochondrial fractions from A549 and A549/DDP cells (Figure 1(d), Table S3). Using the mitochondrial housekeeping gene MT-ND5 as an internal reference, RT-qPCR analysis confirmed that H19 was the most significantly up-regulated nucleus-encoded mitochondrial lncRNA in A549/DDP cells (Figure 1(e)), leading to its selection for subsequent functional characterization.

### Mitochondrial localization of H19

**Figure 2.**
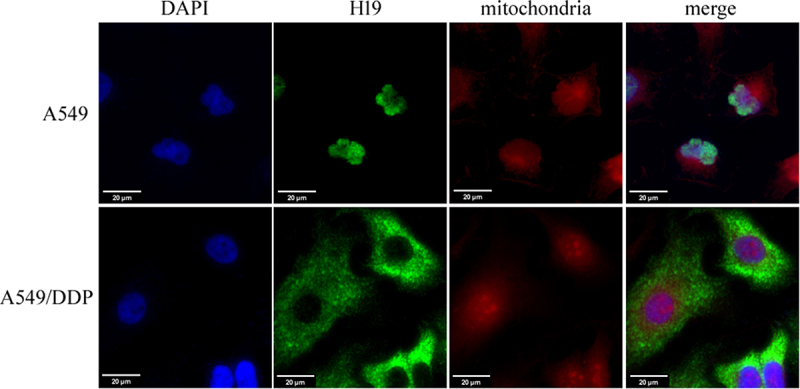
Mitochondrial localization of H19. The H19 probe labeled H19 (green) and MitoTracker were used as mitochondrial markers (red). dapi was used for nuclear staining (blue). Images were obtained using confocal microscopy. Results represent three independent experiments. Bars, 20 μm.

The subcellular localization of H19 was assessed using fluorescence in situ hybridization (FISH). In A549 cells, the H19 signal predominantly overlapped with DAPI-stained nuclei. Conversely, in A549/DDP cells, we observed pronounced colocalization between H19 and MitoTracker-labeled mitochondria (Figure 2). These results indicated that while H19 is primarily nuclear in parental A549 cells, it translocated to mitochondria in drug-resistant cells, suggesting its role as a nucleus-to-mitochondria lncRNA (ntmtlncRNA) potentially involved in mediating DDP resistance in A549/DDP cells.

### H19 silencing enhances DDP sensitivity

**Figure 3.**
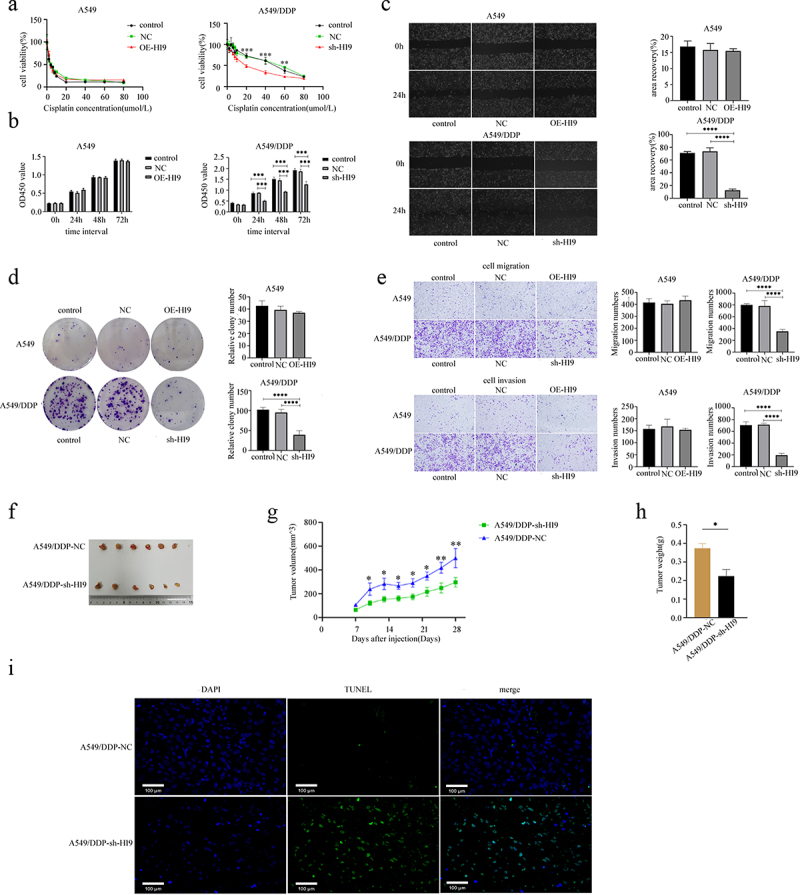
Knockdown of H19 enhances DDP sensitivity. (a) CCK8 assays measured cell viability across various DDP concentrations (0–80 µmol/L). Results represent three independent experiments and are expressed as SD ± mean (bars). Differences in means between the two samples were analysed by the nonparametric rank sum test. (b) CCK8 assessed cell proliferation in each group. Results represent three independent experiments and are expressed as SD ± mean (bars). Differences in means between the two samples were analysed by the multifactor anova. (c) Scratch assays evaluated cell migration and healing. Results represent three independent experiments and are expressed as SD ± mean (bars). Differences in means between the two samples were analysed by the one-way anova. (d) Clonogenic assays determined cell cloning efficiency. Results represent three independent experiments and are expressed as SD ± mean (bars). Differences in means between the two samples were analysed by the one-way anova. (e) Transwell assays tested cell migration and invasion, using 600 µL of drug-containing medium in the lower chamber for 24 hours. Results represent three independent experiments and are expressed as SD ± mean (bars). Differences in means between the two samples were analysed by the one-way anova. (f) Tumor sizes in nude mice at 28 days. (g) Tumor growth curves for each group. Results represent three independent experiments and are expressed as SD ± mean (bars). Differences in means between the two samples were analysed by the one-way anova. (h) Average weight of subcutaneous tumors in nude mice at 28 days. Results represent three independent experiments and are expressed as SD ± mean (bars). Differences in means between the two samples were analysed by the t-test. (i) Tunel staining in nude mouse models, with dapi for nuclear staining (blue) and tunel for apoptotic cells (green). Results represent three independent experiments. Bars, 100 μm.

To investigate the functional role of H19 in mediating DDP resistance in LUAD, we generated A549 cells with stable H19 overexpression and A549/DDP cells with H19 knockdown (Figure S2). CCK-8 assays across a range of DDP concentrations (0–80 μmol/L) demonstrated that H19 overexpression did not significantly alter the viability of OE-H19 A549 cells, whereas H19 silencing substantially reduced the viability of A549/DDP cells compared to controls (Figure 3(a)). Further functional characterization revealed that H19 overexpression had minimal impact on the proliferative, migratory, and invasive capacities of A549 cells. In contrast, H19 knockdown significantly attenuated these malignant behaviors in A549/DDP cells (Figure 3(b-e)). In xenograft models, mice implanted with sh-H19 A549/DDP cells exhibited markedly slower tumor growth and reduced final tumor weights compared to controls following DDP treatment (5 mg/kg administered every three days from day 7 post-implantation) (Figure 3(f-i)). These findings collectively demonstrated that H19 contributed to DDP resistance in LUAD and that its suppression could restore chemosensitivity to DDP-based regimens.

### H19 modulates mitochondrial function to reverse DDP resistance

**Figure 4.**
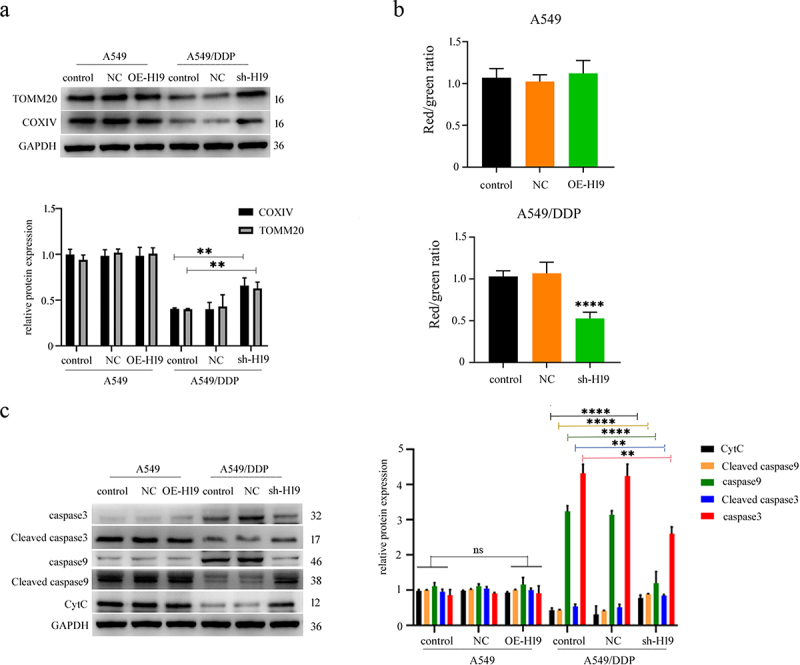
H19 modulates mitochondrial function to reverse DDP resistance. Western blot detection of the expression of mitochondrial proteins cox IV and TOMM20. Results represent three independent experiments and are expressed as SD ± mean (bars). Differences in means between the two samples were analyzed by the one-way anova. (b) JC-1 staining assay to detect the changes in the mitochondrial membrane potential, by using a fluorescence spectrophotometer for detection. Results represent three independent experiments and are expressed as SD ± mean (bars). Differences in means between the two samples were analysed by the one-way anova. (c) Western blot detection of the difference in the expression of mitochondrial apoptotic proteins and CytC release. Results represent three independent experiments and are expressed as SD ± mean (bars). Differences in means between the two samples were analysed by the multifactor anova. **p < 0.01; ***p < 0.001; ****p < 0.0001.

To further investigate how H19 modulates mitochondrial function to promote DDP resistance in LUAD, we assessed mitochondrial protein expression, membrane potential (MMP), and the mitochondrial apoptotic pathway. Western blot analysis revealed that the expression levels of key mitochondrial proteins COXIV and TOMM20 were significantly decreased in A549/DDP cells compared to parental A549 cells. While H19 overexpression in A549 cells did not alter their expression, H19 knockdown in A549/DDP cells substantially restored both proteins to near-normal levels (Figure 4(a)). JC-1 staining demonstrated that H19 silencing significantly reduced MMP in A549/DDP cells – an established indicator of early apoptosis – whereas H19 overexpression had no effect on MMP in A549 cells (Figure 4(b)). This suggests that H19 depletion compromises mitochondrial integrity in resistant cells, thereby facilitating cell death initiation. The observed MMP disruption likely promotes the release of pro-apoptotic factors such as cytochrome c (CytC), which in turn activates downstream apoptotic signaling [26]. Consistent with this mechanism, we detected enhanced cleavage of caspase-3 and caspase-9, along with increased CytC levels, in sh-H19 A549/DDP cells following DDP treatment, while OE-H19 A549 cells showed no such changes (Figure 4(c)). These findings collectively indicated that H19 sustained DDP resistance by preserving mitochondrial function and suppressing apoptosis, and that targeting H19 could reverse this resistance by restoring mitochondrial-mediated cell death pathways.

### H19 silencing suppresses mitochondrial autophagy activity in A549/DDP cells

**Figure 5.**
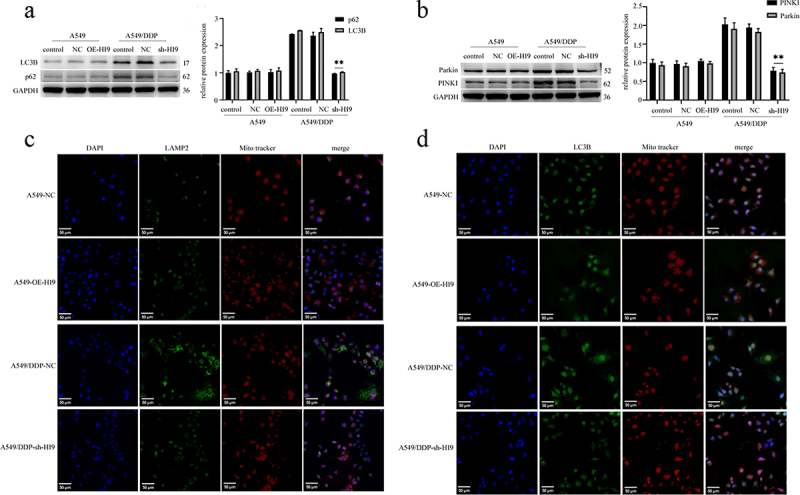
H19 knockdown suppresses mitochondrial autophagy activity in A549/DDP cells. (a) Expression of autophagy proteins LC3B and p62 detected by western blot. Results represent three independent experiments and are expressed as SD ± mean (bars). Differences in means between the two samples were analysed by the one-way anova. (b) Expression of mitochondrial autophagy proteins PINK1 and Parkin. Results represent three independent experiments and are expressed as SD ± mean (bars). Differences in means between the two samples were analysed by the one-way anova. (c) Lysosome-associated membrane glycoprotein 2 and mitochondrial cotransfection, results represent three independent experiments. Scale bars, 50 μm. (d) Autophagy proteins LC3B and mitochondrial cotransfection, results represent three independent experiments. Scale bars, 50 μm. LAMP2 marks lysosomes (green), LC3B as an autophagic protein (green), and MitoTracker was used as mitochondrial markers (red), dapi was used for nuclear staining (blue). Images were obtained using confocal microscopy. **p < 0.01.

Our data indicated that autophagic activity was enhanced in drug-resistant cells, as evidenced by significantly elevated levels of the autophagy-related proteins LC3B and p62 in A549/DDP cells compared to A549 cells. Silencing H19 in A549/DDP cells effectively reduced the expression of both LC3B and p62, whereas H19 overexpression in A549 cells failed to induce significant alterations in these markers (Figure 5(a)). Furthermore, key mitophagy regulators PINK1 and Parkin were substantially upregulated in A549/DDP cells, but this increase was reversed following H19 knockdown. Conversely, H19 overexpression in sensitive cells did not markedly affect PINK1 or Parkin expression (Figure 5(b)). Dual-color immunofluorescence staining further demonstrated enhanced colocalization of mitochondrial (MitoTracker) and lysosomal (LAMP2) signals in A549/DDP cells, indicating accelerated mitophagic flux. This enhanced colocalization was substantially diminished upon H19 silencing but remained unaffected by H19 overexpression in A549 cells (Figure 5(c, d)). Collectively, these findings suggest that H19 promotes mitophagy in A549/DDP cells as a cytoprotective mechanism that supports cell survival under chemotherapeutic stress. Suppression of H19 expression effectively inhibits this protective mitophagic response, thereby sensitizing resistant cells to DDP-induced cell death, while exerting limited effect on the parental A549 cell line.

### Inhibition of mitochondrial autophagy reverses DDP resistance in A549/DDP cells

**Figure 6.**
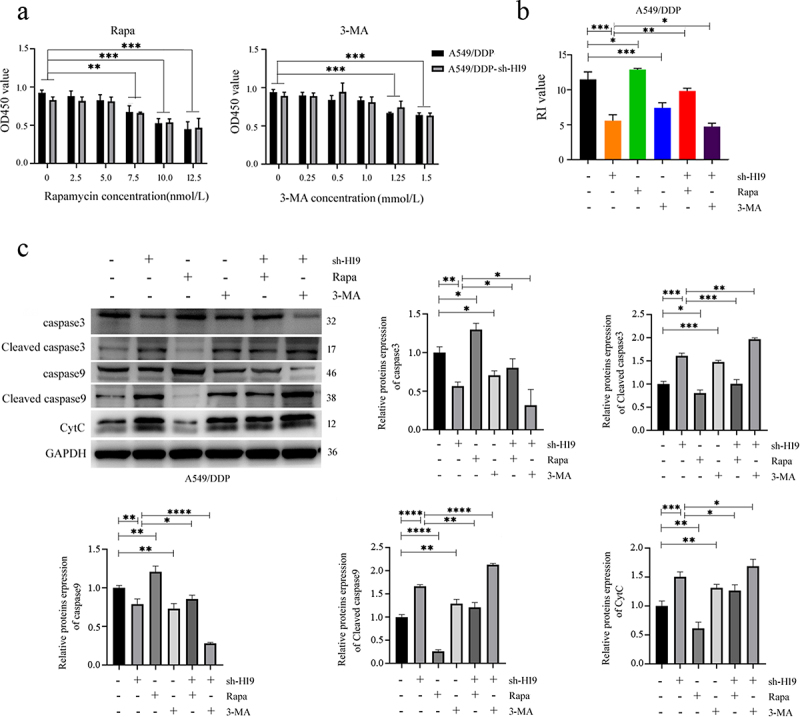
Inhibition of mitochondrial autophagy reverses DDP resistance in A549/DDP cells. (a) CCK8 screening of 3-MA and Rapa drug concentrations. Results represent three independent experiments and are expressed as SD ± mean (bars). Differences in means between the two samples were analysed by the one-way anova. (b) Changes in drug resistance index of lung adenocarcinoma resistant cells after knockdown of H19 and interference with autophagy. Results represent three independent experiments and are expressed as SD ± mean (bars). Differences in means between the two samples were analysed by the multifactor anova. (c) Changes in mitochondrial apoptotic proteins of lung adenocarcinoma resistant cells after knockdown of H19 and interference with autophagy, the concentration of DDP given to each group of cells was 2 mg/L. Results represent three independent experiments and are expressed as SD ± mean (bars). Differences in means between the two samples were analysed by the multifactor anova. **p < 0.01; ***p < 0.001; ****p < 0.0001.

Since H19 overexpression showed minimal effects on A549 cells, we focused subsequent investigations on A549/DDP cells. Using CCK-8 assays, we first determined the optimal working concentrations for the autophagy activator rapamycin (Rapa, 5 nmol/L) and inhibitor 3-methyladenine (3-MA, 1 μmol/L) (Figure 6(a)). In A549/DDP cells, Rapa treatment increased the resistance index, whereas both 3-MA and H19 silencing significantly reduced it, indicating that either autophagy inhibition or H19 knockdown can attenuate DDP resistance. Notably, in H19-silenced A549/DDP cells, Rapa partially restored DDP resistance, while the combination of H19 silencing and 3-MA produced a synergistic sensitizing effect (Figure 6(b)). Following DDP pretreatment, Rapa reduced levels of caspase-3, caspase-9, and CytC, thereby suppressing apoptosis, while 3-MA increased these pro-apoptotic proteins. H19 silencing similarly elevated these apoptosis markers, and this effect was further enhanced by 3-MA co-treatment but attenuated by Rapa (Figure 6(c)). These results demonstrate that H19 promotes DDP resistance through enhancing mitophagy, while its silencing restores chemosensitivity by suppressing this protective mechanism.

### Re-localization of H19

**Figure 7.**
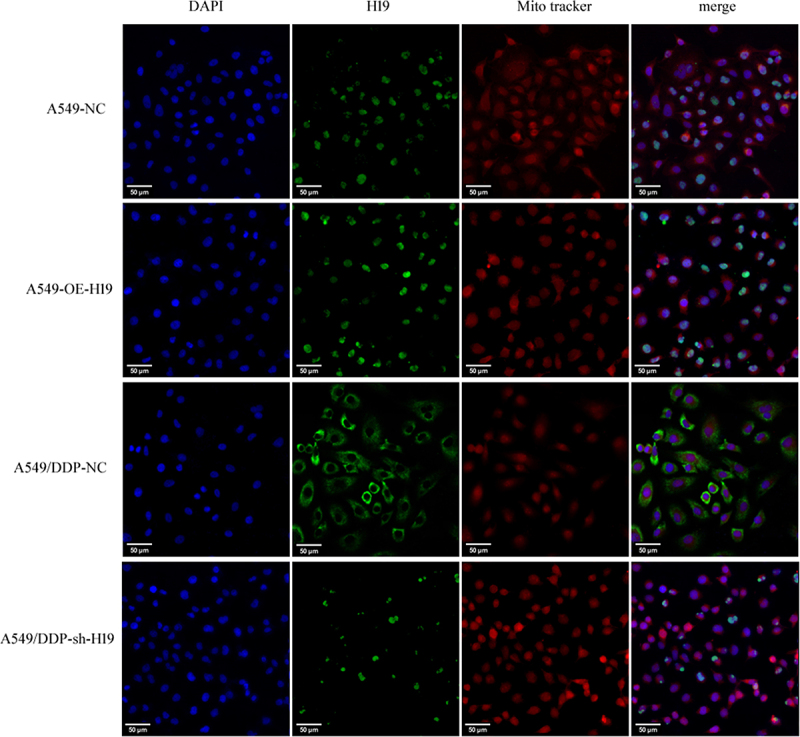
Localization of H19 in mitochondria. The H19 probe labeled H19 (green) and MitoTracker were used as mitochondrial markers (red). dapi was used for nuclear staining (blue). Images were obtained using confocal microscopy. Results represent three independent experiments. Bars, 50 μm.

To further validate the subcellular distribution patterns of H19, we performed FISH assays across four experimental cell groups. The results corroborated our initial observations: H19 was predominantly localized in the nucleus of A549 cells, while in A549/DDP cells it primarily accumulated in mitochondria. Notably, H19 silencing in A549/DDP cells substantially reduced its mitochondrial presence. Conversely, H19 overexpression in A549 cells did not alter its nuclear localization pattern. These findings reinforce the role of H19 as a mitochondria-associated lncRNA (ntmtlncRNA) specifically in chemoresistant A549/DDP cells, while demonstrating its limited functional impact in the parental A549 cell line (Figure 7).

## Discussion

Mitochondria possess their own genetic material, mitochondrial DNA (mtDNA), which operates semi-autonomously from nuclear DNA. Unlike nuclear DNA, mtDNA lacks robust histone protection and efficient repair mechanisms. Its location near the electron transport chain on the inner mitochondrial membrane further renders it highly susceptible to reactive oxygen species (ROS)-induced damage [27–29]. Epidemiological evidence from a prospective cohort study suggests that elevated mtDNA copy number may correlate with increased lung cancer risk [30]. Conversely, experimental studies demonstrate that reducing mtDNA content enhances tumor cell sensitivity to chemotherapeutic agents including DDP and docetaxel [31]. While mitochondrial dysfunction and mtDNA mutations are common features in cancer cells, mitochondrial energy production and functional capacity are not completely abolished. Instead, cells activate compensatory mechanisms – particularly through mitochondrial-nuclear communication. Mitochondrial dysfunction triggers “retrograde signaling” to the nucleus, while nuclear “anterograde regulation” continuously modulates mitochondrial activity [32,33]. This bidirectional signaling axis creates an integrated regulatory network that maintains cellular homeostasis and enables adaptation to various stress conditions [34–36].

Recent studies have established mitochondria-associated lncRNAs as crucial mediators of mitochondrial-nuclear communication [25,37]. These regulatory molecules can be categorized into two groups: mtDNA-encoded lncRNAs (mtlncRNAs) and nucleus-encoded mitochondria-associated lncRNAs (ntmtlncRNAs). Early identified mtlncRNAs, including Sense Non-Coding Mitochondrial RNA (SncmtRNA) and Antisense Non-Coding Mitochondrial RNAs (ASncmtRNAs), participate in nuclear transport and retrograde signaling. Notably, ASncmtRNA knockdown induces apoptosis in cancer cells by suppressing survivin expression while minimally affecting normal cells, highlighting their therapeutic potential [38,39]. Meanwhile, certain ntmtlncRNAs interact directly with mitochondrial proteins to regulate metabolic processes and translation. For instance, SAMMSON associates with the mitochondrial surface protein p32 to maintain mitochondrial integrity; its inhibition shifts energy production from oxidative phosphorylation to glycolysis and suppresses melanoma proliferation [40,41]. Our investigation revealed substantially different H19 expression patterns between A549/DDP cells and their parental counterparts. FISH analysis demonstrated that while H19 primarily localizes to the nucleus in A549 cells, it undergoes mitochondrial translocation in A549/DDP cells. Based on these findings, we propose that H19 functions as a ntmtlncRNA that potentially modulates DDP resistance in lung adenocarcinoma through mitochondrial signaling pathways.

Previous studies have implicated H19 in mediating DDP resistance across various malignancies through diverse mechanisms, including miR-107/HMGB1-mediated autophagy in laryngeal squamous cell carcinoma and glutathione metabolism in high-grade serous ovarian cancer. Hypoxic conditions have been shown to upregulate H19 expression, thereby promoting DDP resistance in NSCLC cells [42–44]. Our in vitro experiments demonstrated that H19 knockdown in A549/DDP cells significantly suppressed proliferative capacity, delayed wound closure, and impaired migratory, invasive, and clonogenic potentials – findings corroborated by our in vivo observations. At the molecular level, H19 depletion in resistant cells upregulated mitochondrial markers COXIV and TOMM20, reduced mitochondrial membrane potential, and enhanced expression of apoptotic mediators (caspase-3, caspase-9, and CytC), indicating H19’s critical role in regulating mitochondrial integrity and apoptotic signaling. Importantly, these phenotypic and molecular alterations were not observed in parental A549 cells, suggesting that H19’s functional impact is highly dependent on cellular context.

Mitophagy serves as an essential mitochondrial quality control mechanism that selectively eliminates damaged mitochondria under stress conditions [45,46]. The PINK1/Parkin pathway plays a central role in this process, where mitochondrial depolarization triggers PINK1 stabilization and Parkin recruitment, ultimately leading to autophagic clearance of compromised organelles [47,48]. Chemotherapeutic agents frequently induce mitochondrial oxidative stress and dysfunction, and enhanced elimination of damaged mitochondria through mitophagy has been increasingly recognized as a contributor to chemoresistance in malignancies [49]. Emerging evidence indicates that H19 can regulate mitophagy via the PINK1/Parkin axis in pathological contexts such as obesity-related cardiac dysfunction [50]. Nevertheless, the specific involvement of H19 in modulating mitophagy within lung cancer, particularly in the context of chemoresistance, remains to be fully elucidated.

Our experimental results demonstrated that H19 knockdown in chemoresistant cells significantly reduced the expression of autophagy markers (LC3B, p62) and key mitophagy regulators (PINK1, Parkin), accompanied by diminished mitochondrial colocalization with lysosomal and LC3B signals. These findings establish H19’s role in promoting mitophagy in A549/DDP cells. A notable strength of our investigation lies in the combined application of genetic approaches (H19 knockdown and overexpression) with pharmacological modulators (rapamycin and 3-MA), providing complementary evidence for H19’s function in mitophagy regulation. Consistent with genetic manipulation, pharmacological interventions with rapamycin and 3-MA further validated that H19 depletion enhances DDP sensitivity. Immunofluorescence analysis confirmed H19’s mitochondrial translocation specifically in A549/DDP cells, a redistribution phenomenon not observed upon H19 overexpression in parental A549 cells, underscoring H19’s context-dependent role as a mitochondrial function modulator in chemoresistant cells.

While our findings reveal a novel connection between H19 and mitophagy-mediated DDP resistance, several limitations should be considered when interpreting these results. First, the absence of mitochondrial-specific H19 knockdown approaches represents a potential methodological constraint. Our current experiments employed pan-cellular H19 depletion, which prevents definitive distinction between the contributions of mitochondrial versus nuclear H19 pools. This approach may overestimate H19’s direct mitochondrial functions, as nuclear H19 could indirectly influence mitophagy through transcriptional regulation. The minimal phenotypic impact of H19 overexpression in parental cells further underscores the context-dependent nature of H19’s functions, necessitating validation across additional LUAD models with diverse genetic backgrounds. Second, the exclusive reliance on in vitro cell line models, while valuable for mechanistic insights, limits physiological relevance. Although A549/DDP cells provide a validated model of acquired resistance, they lack the complex tumor microenvironment and immune interactions present in vivo. This simplification may lead to underestimation of H19’s systemic effects on mitophagy, particularly in tumor-stroma communication. Future investigations employing patient-derived xenografts or organoid models will be crucial for validating H19’s role under more physiologically relevant conditions.

While our study demonstrates H19’s involvement in DDP resistance through mitophagy regulation, we recognize that this represents only one aspect of its multifaceted functions in tumor biology. The complex nature of H19 as a pluripotent lncRNA suggests it likely participates in additional molecular pathways beyond the mitochondrial mechanisms explored here. Future research should explore H19’s potential roles in other critical processes such as metabolic reprogramming, tumor-stroma interactions, and integration with established resistance pathways to fully elucidate its therapeutic relevance in lung adenocarcinoma.

## Conclusion

Our findings establish H19 as a critical mediator of mitochondrial-nuclear communication in A549/DDP cells. By demonstrating its role in regulating mitochondrial quality control and mitophagy, this work supports H19 as a promising therapeutic target for overcoming DDP resistance and improving treatment outcomes in lung adenocarcinoma.

## Supplementary Material

Supplemental Material

ARRIVE guidelines.pdf

Ethics Approval Translation.pdf

## Data Availability

The datasets generated or analyzed in this study can be obtained from the corresponding author upon reasonable request.
