# Supplementary material for: Mitophagy suppression via lncRNA H19 silencing: a novel strategy to overcome cisplatin resistance in lung adenocarcinoma
Source: Cell Cycle. 2025 Nov 5;24(21-24):670–86. doi: 10.1080/15384101.2025.2581634 (PMC12918326; doi:10.1080/15384101.2025.2581634)
Supplement: Supplemental Material [file KCCY_A_2581634_SM3743.docx]

**Table S1.** The expected size of the amplicon in bp, the annealing temperatures, GenBank Accession numbers

| Genes | Forward primer (5'-3') | Reverse primer (5'-3') | bp | annealing temperatures（℃） | Id |
| --- | --- | --- | --- | --- | --- |
| MT-ND5 | CCTATTCCAACTGTTCATCGGCT | ATTGCTTGAATGGCTGCTGTG | 108 | 62.4 | 4540 |
| GAPDH | GGAAGCTTGTCATCAATGGAAATC | TGATGACCCTTTTGGCTCCC | 168 | 62.4 | 2597 |
| H19 | CTCCCAGAACCCACAACATG | CAGTGGTTGAAAGTGCAGCATA | 116 | 58.46 | 283120 |
| LINC00998 | TGCCTGTTGTGGAAGCAGTAGAA | GCACAAGGCAGGCAAGACCA | 82 | 63.33 | 401397 |
| MMP24-AS1 | ACGAVGTGCGCTTCCTCAT | AGACAAGGCAGGCAAGAACCA | 106 | 62.28 | 101410538 |
| LINC01133 | AACCTTTGCTCCAACTTTCTCCT | CTCTTTACCTCCTCCCAACCATT | 217 | 60.37 | 100505633 |
| FLG-AS1 | TGTCCCTCACTGTCCCTGTCCT | GTCTCCCTCTGTGACTTCCCTCT | 223 | 64.47 | 339400 |
| LINC00707 | GACTTTACTGGCTTTCTTGCTCC | GACCTTAACCTTCCATCATCCCT | 118 | 59.81 | 100507127 |

**Table S2.** The exact sequence of the H19 probe

| H19 | probe1 | TCTTTGATGTTGGGCTGATGAGGTC |
| --- | --- | --- |
|  | probe2 | CTAGCTTCACCTTCCAGAGCCGATT |
|  | probe3 | AGGTAGTGCAGTGGTTGTAAAGTGC |
|  | probe4 | TCATTTCCAAGCTAGAGGGTTTTGT |
|  | probe5 | CTTGAGCTGGGTAGCACCATTTCT |

**Table S3.** Top 10 differentially expressed lncRNAs by mitochondrial high-throughput sequencing

| Gene | baseMean | Log2FoldChange | IfcSE | Stat | pvalue | padj |
| --- | --- | --- | --- | --- | --- | --- |
| LINC00998 | 416.7507 | 2.190073 | 0.159004 | 13.7737 | 3.67E-43 | 3.29E-41 |
| H19 | 154.8937 | 4.213414 | 0.312151 | 13.49801 | 1.61E-41 | 1.33E-39 |
| MMP24-AS1 | 957.7212 | 2.414926 | 0.192301 | 12.55805 | 3.59E-36 | 2.27E-34 |
| LINC01133 | 126.3372 | 4.49873 | 0.364545 | 12.34067 | 5.47E-35 | 3.27E-33 |
| FLG-AS1 | 119.5876 | 6.831105 | 0.585481 | 11.66751 | 1.87E-31 | 9.28E-30 |
| LINC00707 | 90.45467 | 3.111102 | 0.293313 | 10.60677 | 2.77E-26 | 9.81E-25 |
| LINC01116 | 111.6401 | 2.601923 | 0.259307 | 10.03415 | 1.08E-23 | 3.24E-22 |
| RP11-785H5.1 | 251.3978 | 1.62875 | 0.162762 | 10.00693 | 1.42E-23 | 4.24E-22 |
| RP11-5407.1 | 68.24229 | 3.57922 | 0.380455 | 9.40774 | 5.07E-21 | 1.26E-19 |
| AC006262.4 | 46.29249 | 5.245995 | 0.625875 | 8.381854 | 5.21E-17 | 9.17E-16 |


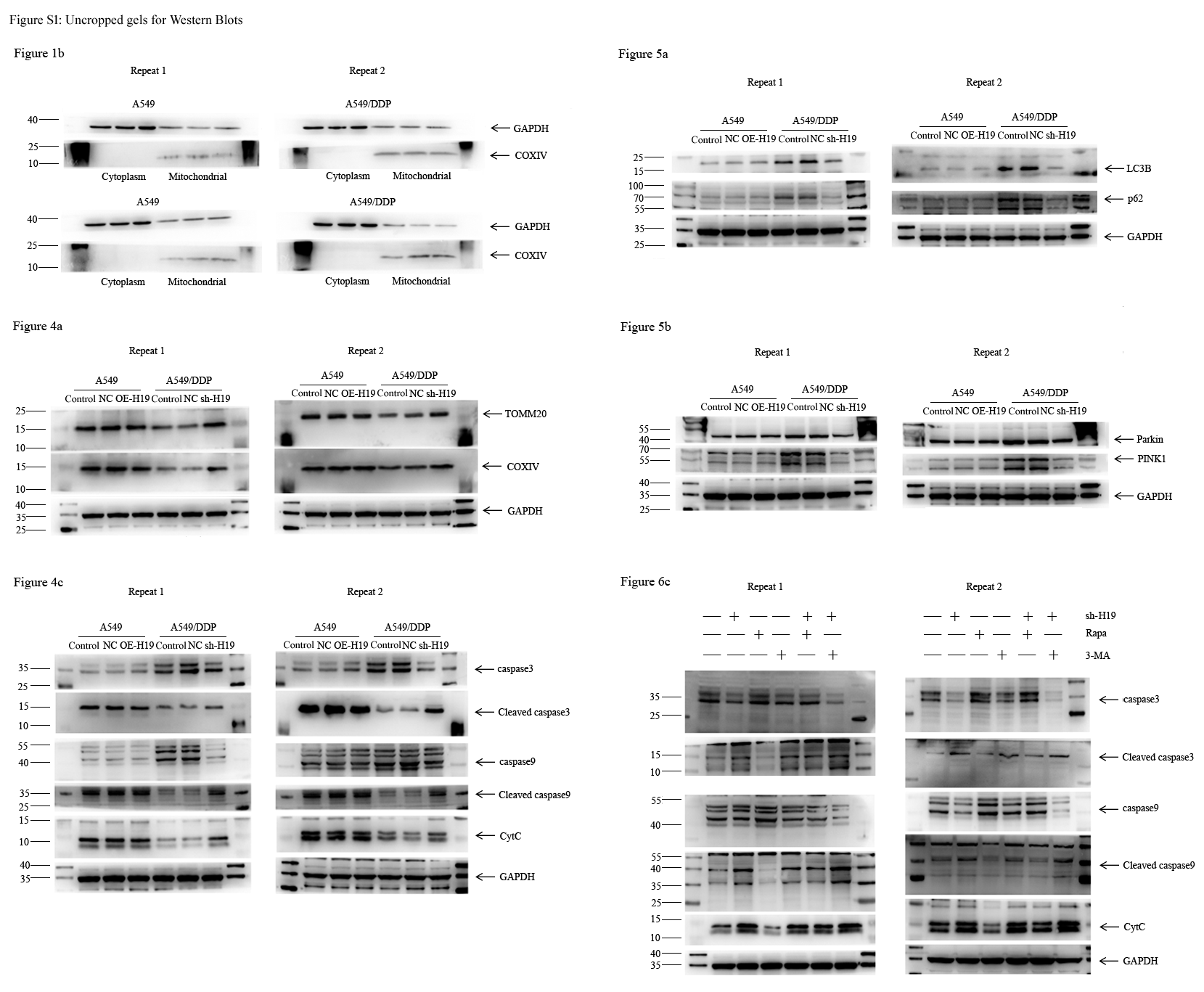


**Figure S1**: Gel images for western blot.


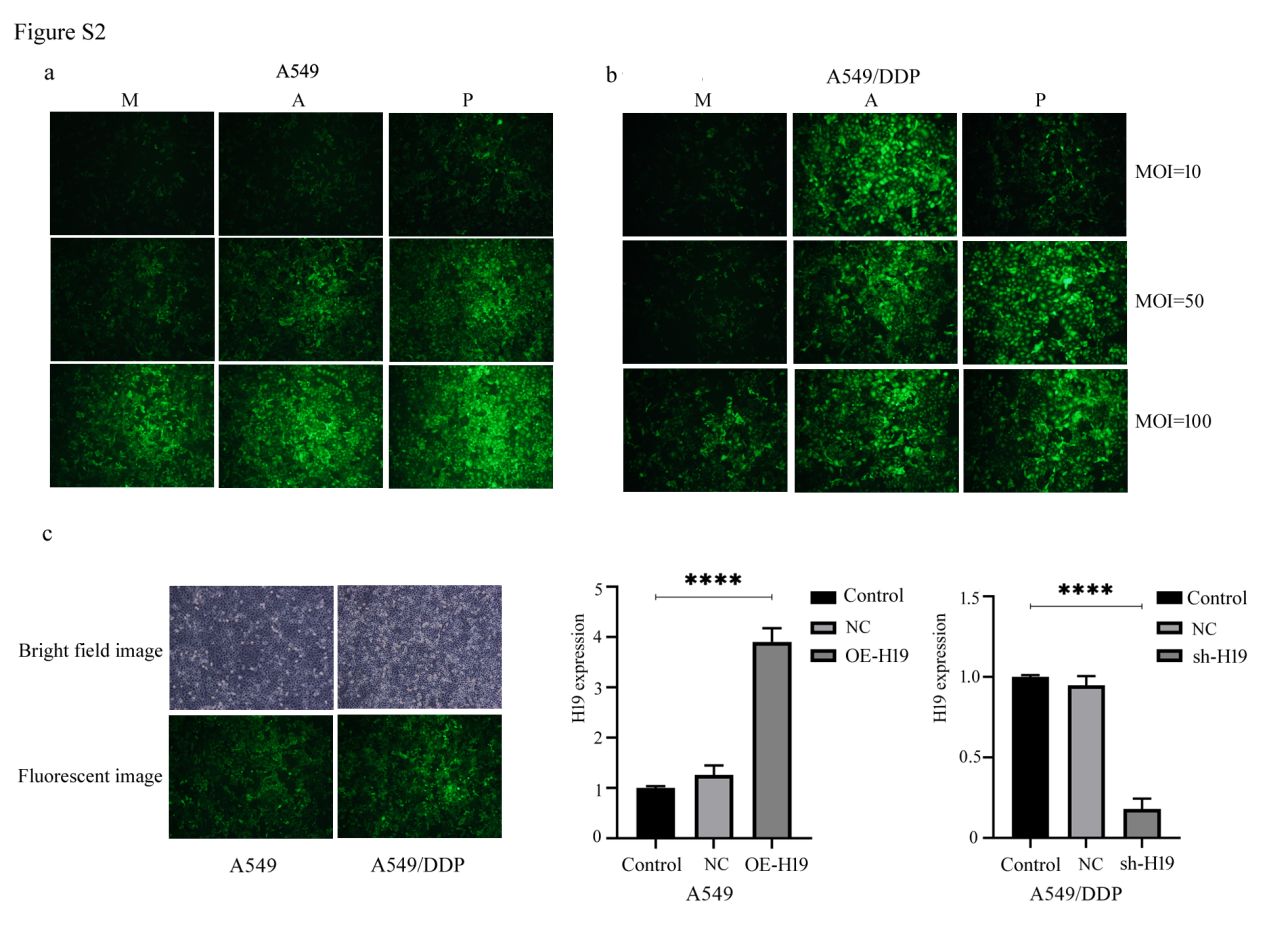


**Figure S2.** Construction of lentivirus infected cell stable strain. (A) Pre-experiment to explore the optimal conditions for lentiviral infection. (B) Status of stabilized cell lines under bright field and fluorescence. (C) RT-qPCR to verify the efficiency of lentiviral infection. Results represent three independent experiments and are expressed as SD ± mean (bars). Differences in means between the two samples were analysed by the one-way ANOVA. *****P* < 0.0001.
