# Supplementary material for: Mitophagy suppression via lncRNA H19 silencing: a novel strategy to overcome cisplatin resistance in lung adenocarcinoma
Source: Cell Cycle. 2025 Nov 5;24(21-24):670–86. doi: 10.1080/15384101.2025.2581634 (PMC12918326; doi:10.1080/15384101.2025.2581634)
Supplement: Ethics Approval Translation.pdf [file KCCY_A_2581634_SM9439.pdf]

Experimental Animal Ethical Review Report

Our school intends to carry out a scientific research project named "The Mechanism of mtlncRNATVAS5 Mediated Cisplatin Resistance in Lung Adenocarcinoma through the Mitochondrial [Ca2+]m Pathway". Since this project involves animal experiments, the Experimental Animal Ethics Committee of our school has reviewed the relevant experimental animal ethics issues of this project.

Project Information: General Program of the National Natural Science Foundation of China.
Project Name: The Mechanism of mtlncRNATVAS5 Mediated Cisplatin Resistance in Lung Adenocarcinoma through the Mitochondrial [Ca2+]m Pathway.
Undertaking Unit: Xuzhou Medical University, Project Responsible Person: Sun Sanyuan
Research Start and End Dates: January 2021 - December 2024
Main Contents of Animal Research (Fill in according to the subject situation):
Construct an overexpression plasmid vector of TVAS5 to transfect A549 cells, and use Antisense oligonucleotides (ASOs) to down-regulate the expression of TVAS5 in A549/DDP cells, and then inoculate BALB/C-nu/nu nude mice. When the tumor grows to about 5 mm in diameter, treat with cisplatin (5mg/kg), inject intraperitoneally once every 3 days, for a total of 3 injections.
Since the administration, measure the tumor diameter with a caliper every 3 days, calculate the average volume of the tumor mass (mm3), and draw a growth curve.
Review and Comment Opinions
After deliberation by the Experimental Animal Ethics Committee of our school, the experimental design and implementation plan of this research fully consider the principles of safety and fairness. The research content does not pose harm and risk to the experimental animals, nor does it pose harm to the researchers and other people. There is no conflict of interest in the research content and results.
Conclusion
In this research, the experimental content meets the requirements of animal ethics, there are no potential risks, and it is agreed that the work of this research be carried out according to the plan.

Xuzhou Medical University
Experimental Animal Ethics Committee
March 10, 2020
